# Rapid Plasmid-Free Generation of Recombinant Positive-Strand RNA Viruses That Use IRES-Mediated Translation Using an Expansion of the Circular Polymerase Extension Reaction (CPER)

**DOI:** 10.21769/BioProtoc.5275

**Published:** 2025-04-20

**Authors:** Hirotaka Yamamoto, Tomokazu Tamura, Takausuke Fukuhara

**Affiliations:** 1 Department of Microbiology and Immunology, Faculty of Medicine, Hokkaido University, Sapporo, Japan; 2 Institute for Vaccine Research and Development (IVReD), Hokkaido University, Sapporo, Japan; 3 One Health Research Center, Hokkaido University, Sapporo, Japan; 4 Department of Virology, Faculty of Medical Sciences, Kyushu University, Fukuoka, Japan; 5 Laboratory of Virus Control, Research Institute for Microbial Diseases, Osaka University, Suita, Japan; 6 AMED-CREST, Japan Agency for Medical Research and Development (AMED), Tokyo, Japan

**Keywords:** Reverse genetics, Positive-strand RNA viruses, Recombinant viruses, Circular polymerase extension reaction (CPER), IRES-mediated translation

## Abstract

Reverse genetics systems in virology are technologies used to generate recombinant viruses, enabling the manipulation of viral genes. Recombinant viruses facilitate the investigation of pathogenesis and the development of antivirals. In studies of positive-sense single-stranded RNA (ssRNA) viruses, a reverse genetics approach typically uses infectious viral cDNA clones derived from bacterial artificial chromosomes and plasmids or from the in vitro ligation of viral cDNA fragments. However, these methods are time-consuming, involve complex procedures, and do not always successfully generate recombinant viruses. Possible reasons for unsuccessful outcomes include i) viral sequences exhibiting toxicity in bacterial systems, ii) the duplication of viral genes observed in some strains, complicating the acquisition of correct cDNA clones, and iii) certain cell lines being highly susceptible to infection but difficult to transfect with nucleotides. For these reasons, a simple and rapid reverse genetics system is needed to accelerate research on ssRNA viruses. The circular polymerase extension reaction (CPER) method offers a solution by eliminating the need for molecular cloning in bacteria, enabling the generation of recombinant viruses over a shorter timeframe. This method has been widely adopted for the study of ssRNA viruses, including SARS-CoV-2 and flaviviruses. Recently, we expanded the CPER method for ssRNA viruses using internal ribosome entry site (IRES)-mediated translation. This protocol details the experimental procedures, using bovine viral diarrhea virus as an example—one of the most challenging viruses for generating viral cDNA clones because of the factors listed above.

Key features

• Rapid generation of recombinant positive-strand RNA viruses.

• The CPER method eliminates the need for molecular cloning in bacteria, enabling the rapid generation of recombinant viruses.

• The CPER method for ssRNA viruses enables efficient translation of viruses using IRES by incorporating the gene cassette of RNA Pol-I promoters and terminators.

## Graphical overview


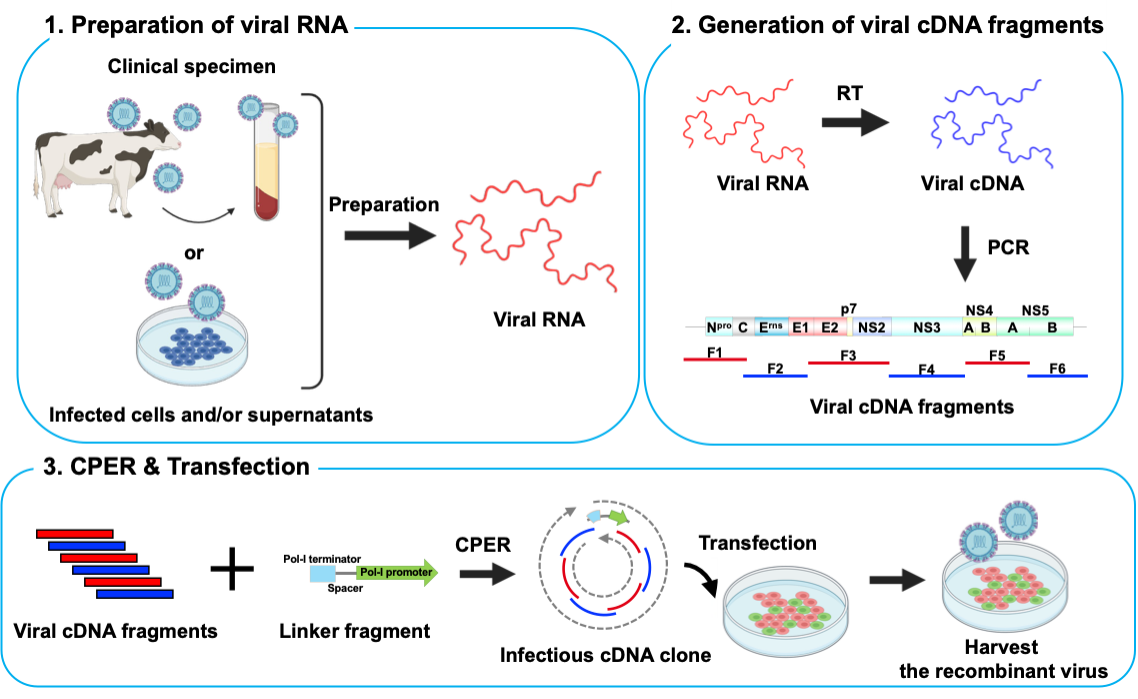


## Background

In virology, the reverse genetics system is a technology for generating recombinant viruses that enables the manipulation of viral genes. Using this system, we can define the function of viral genes/proteins and investigate the mechanisms of viral pathogenicity [1]. To date, infectious viral cDNA clones from bacterial artificial chromosomes and plasmids or from in vitro ligation of viral cDNA fragments have been used for reverse genetics in studies on positive-sense single-stranded RNA (ssRNA) viruses [2–5]. Although these methods allow for the generation of recombinant viruses, they are time-consuming and involve complicated procedures. Thus, a simple and efficient reverse genetics system is required, particularly in the context of pandemics caused by emerging/re-emerging ssRNA viruses.

A method for the generation of recombinant ssRNA viruses using circular polymerase extension reaction (CPER) has been developed [6,7]. In this method, full-length viral cDNA fragments with overlapping ends and an RNA Pol-II promoter-encoding linker are assembled into a circular genome by DNA polymerase and are directly transfected into cells to recover infectious viruses. This technology was applied to severe acute respiratory syndrome coronavirus 2 (SARS-CoV-2), which possesses one of the largest genomes (~30 kb) among the ssRNA viruses, enabling the rapid generation of recombinant viruses. It also enabled the rapid characterization of SARS-CoV-2 variants of concern and variants of interest after their emergence [8–12].

Flaviviruses and SARS-CoV-2 perform translation in a cap-dependent manner, whereas certain ssRNA viruses, such as hepatitis C virus (HCV) and bovine viral diarrhea virus (BVDV), perform cap-independent internal ribosome entry site (IRES)-mediated translation. The CPER method, optimized for efficient capping after viral RNA transcription, may not be directly applicable to ssRNA viruses that use IRES-mediated translation. Therefore, we adapted the CPER method using a gene cassette containing an RNA Pol-I promoter and terminator sequence that is needed for the efficient translation of RNA viruses [13].

BVDV, now renamed as *Pestivirus bovis*, belongs to the *Pestivirus* genus of the *Flaviviridae* family and possesses ssRNA that undergoes IRES-mediated translation. BVDV is the causative agent of bovine viral diarrhea, which leads to economic losses in agriculture. Generating recombinant BVDV using conventional reverse genetics has proven challenging for three reasons: i) the viral sequence exhibits toxicity in bacteria [14]; ii) gene duplication in some BVDV strains makes it difficult to obtain the correct cDNA clones [15]; and iii) MDBK cells, the most common cell line for BVDV propagation, show high susceptibility but are difficult to transfect with nucleotides [16].

In this protocol, we describe an optimized CPER method for ssRNA viruses using IRES-mediated translation, with BVDV serving as an example. Our method overcomes commonly encountered challenges and successfully produces recombinant viruses over a short timeframe. In addition, we demonstrate the generation of recombinant viruses without the need for isolation from permissive cell cultures, which is useful for the rapid characterization of viral strains, particularly during a pandemic.

## Materials and reagents

**Biological materials**

1. Bovine MDBK cells (ATCC, catalog number: CCL-22)

2. Lenti-X 293T cells (TAKARA BIO, catalog number: 632180)

3. Serum from cattle persistently infected with BVDV (BVDV-1b strain Shihoro/B_6, Hirose et al. [17])

**Reagents**

1. BVDV antibody-free FBS (Japan Bio Serum, catalog number: 621-00535)

2. DMEM (4.5 g/L glucose) with L-glutamine, without sodium pyruvate, liquid (Nacalai Tesque, catalog number: 08459-64)

3. Penicillin-streptomycin mixed solution (Nacalai Tesque, catalog number: 26253-84)

4. PrimeSTAR^®^ GXL DNA polymerase (TAKARA BIO, catalog number: 12292-04)

5. KOD One^®^ PCR master mix (Toyobo, catalog number: 18538-01)

6. 1 kb DNA ladder (TAKARA BIO, catalog number: 3412A)

7. SuperScript^TM^ IV VILO^TM^ master mix (Thermo Fisher Scientific, catalog number: 11756050)

8. Opti-MEM (Thermo Fisher Scientific, catalog number: 07088-54)

9. TransIT^®^-LT1 transfection reagent (Mirus, catalog number: MIR2304)

10. Acetone (Nacalai Tesque, catalog number: 00309-35)

11. D-PBS (-) without Ca and Mg, liquid (Nacalai Tesque, catalog number: 14249-24)

12. Anti-pestiviral NS3 antibody (prepared in-house, see Kameyama et al. [18])

13. Goat anti-mouse IgG Alexa Fluor 488-conjugated secondary antibody (Thermo Fisher Scientific, catalog number: A32723)

14. PureLink^TM^ RNA Mini kit (Thermo Fisher Scientific, catalog number: 12183018A)

15. FastGene Gel/PCR Extraction kit (NIPPON Genetics, catalog number: FG-91202)

16. Primer sets (Fasmac; Table 1)

**Table 1.** Primer sets used for CPER of BVDV-1b

Fragment	Genome size (bp)	Orientation	Nucleotide sequence (5′–3′)
BVDV-1b Fragment 1	1303	Forward	GTATACGAGGTTAGGCAAGTTC
		Reverse	GATTTTCTCTGGCCAGATC
BVDV-1b Fragment 2	2401	Forward	GATCTGGCCAGAGAAAATC
		Reverse	CTCTCTTAGTAGTAGGTATAGTAG
BVDV-1b Fragment 3	2316	Forward	CTACTATACCTACTACTAAGAGAG
		Reverse	CATGTATTGATAGACTGACTCAGCTGC
BVDV-1b Fragment 4	2501	Forward	GCAGCTGAGTCAGTCTATCAATACATG
		Reverse	CTTATCTTCCCTTCAGAGTCCATC
BVDV-1b Fragment 5	2236	Forward	GATGGACTCTGAAGGGAAGATAAG
		Reverse	GTGCTTCTCTGAGTCCAGTAC
BVDV-1b Fragment 6	1652	Forward	GTACTGGACTCAGAGAAGCAC
		Reverse	GGGGCTGTTAAGGGTTTTCCCTAGTC
BVDV-1b Linker fragment	738	Forward	ACCCTTAACAGCCCCCCCCCCCAACTTCGGAGGTCGAC
		Reverse	GAACTTGCCTAACCTCGTATACAATAACCCGGCGGCCCAAAATGC

**Solutions**

1. Maintenance medium (see Recipes)

2. 2% FBS medium (see Recipes)

**Recipes**

**1. Maintenance medium**

**Table:** 

Reagent	Final concentration	Quantity or volume
DMEM	Not applicable	500 mL
BVDV antibody-free FBS	9.1%	50 mL
Penicillin-streptomycin	1%	5 mL

**2. 2% FBS medium**

**Table:** 

Reagent	Final concentration	Quantity or volume
DMEM	Not applicable	500 mL
BVDV antibody-free FBS	2%	10 mL
Penicillin-streptomycin	1%	5 mL

**Laboratory supplies**

1. Collagen-coated microplate 6-well with lid, collagen type I (IWAKI, catalog number: 4810-010N)

2. Collagen-coated microplate 24-well with lid, collagen type I (IWAKI, catalog number: 4820-010)

3. Flat bottom micro tube 1.5 mL (BIO-BIK, catalog number: CF-0150)

4. 0.2 mL 8-strip PCR tube caps with dome top, natural (WATSON BIO LAB, catalog number:137-432C)

## Equipment

1. CO_2_ incubator (PHCbi, model: MCO-170AICUVD)

2. NanoDrop Lite UV-Vis spectrophotometer ND-LITE (Thermo Fisher Scientific, catalog number: 32-1001)

3. Fluorescence microscope (KEYENCE, model: BZ-X810)

## Software and datasets

1. Prism v. 10.2.2 (GraphPad, 7/31/2024)

## Procedure

**A. Preparation of viral RNA**

1. Follow the manufacturer’s protocol to purify total RNA from the serum of cattle persistently infected with BVDV using the PureLink^TM^ RNA Mini kit (see General note 1). Use 100 μL of serum stored at -80 °C.

**B. Generation of viral cDNA fragments**

1. Reverse transcribe total RNA into cDNA using the SuperScript^TM^ IV VILO^TM^ master mix. Mix 16 μL of total RNA sample with 4 μL of SuperScript^TM^ IV VILO^TM^ master mix and heat at 25 °C for 10 min, 50 °C for 10 min, and 85 °C for 5 min.

2. Amplify a total of six viral gene fragments covering the entire viral genome and a linker fragment encoding a Pol-I terminator, spacer, and a Pol-I promoter by KOD One^®^ PCR master mix with gene-specific primer sets (primer sets in Table 1; the recipe of this reaction mixture is in Table 2; thermocycling conditions for the PCR reaction are in Table 3) [19].

**Table 2.** Reaction mixture

Reagent	Volume
cDNA	1 μL
Forward primer (10 μM)	1.5 μL
Reverse primer (10 μM)	1.5 μL
KOD One^®^ PCR master mix	25 μL
H_2_O	21 μL

**Table 3.** Thermocycling conditions for PCR

Step	Temp. (°C)	Duration	No. of cycles
Denaturation	98	10 s	30
Annealing	50	5 s	
Extension	68	40 s	
Final extension	68	4 min	1
Hold	4	∞	-

3. Assess the size of the fragments by gel electrophoresis using a commercial 1 kb DNA ladder as a reference.

4. Follow the manufacturer’s protocol to purify the PCR fragments using the FastGene Gel/PCR Extraction kit.

5. Measure DNA concentration using NanoDrop.

6. Because CPER is based on assembly PCR, the correct joining of fragments via the overlapping regions is critical for making the complete circular cDNA assembly. Thus, use PCR assembly to connect neighboring fragments by PrimeSTAR^®^ GXL DNA polymerase (see Troubleshooting 1).

**C. Circular polymerase extension reaction (CPER)**

1. Mix each gene fragment of BVDV cDNA and the linker fragment at 0.1 pmol per fragment.

2. Assemble all fragments of equal molar amounts using PrimeSTAR^®^ GXL DNA polymerase (the recipe of this reaction mixture is in Table 4; thermocycling conditions for the CPER are detailed in Table 5).

**Table 4.** CPER mixture

Reagent	Volume
Each gene fragment of BVDV cDNA and the linker fragment	0.1 pmol per fragment
dNTP mixture	4 μL
5× PrimeSTAR GXL buffer	10 μL
PrimeSTAR GXL DNA polymerase	1 μL
H_2_O	Up to 50 μL

**Table 5.** Thermocycling conditions for the CPER

Step	Temp. (°C)	Duration	No. of cycles
Initial denaturation	98	2 min	1
Denaturation	98	10 s	20
Annealing	55	15 s	
Extension	68	12 min	
Final extension	68	12 min	1
Hold	4	∞	-

**D. Transfection**

1. Prepare cells.

a. One day before transfection, co-culture MDBK and 293T cells in a 2:1 ratio (see General note 2) in maintenance medium (see Recipes) in a collagen-coated 6-well microplate. Seed cells by plating 7 × 10^5^ cells per well.

b. Upon 80% confluence, replace maintenance medium with 2% FBS medium (see Recipes).

2. Immediately after the CPER, add 25 μL of CPER product to 200 μL of Opti-MEM warmed to 37 °C and mix by pipetting.

3. Add 12 μL of TransIT-LT1^®^ transfection reagent and mix by slow pipetting.

4. Centrifuge at 5,000× *g* for several seconds and incubate for 10 min.

5. Add the whole mixture to the cells and culture in a CO_2_ incubator at 37 °C in a humidified atmosphere with 5% CO_2_.

6. After 4 days of transfection, collect 100 μL of culture supernatant in a flat bottom microtube and store at -80 °C.

**E. Detection of viruses by immunofluorescence staining**

**Figure 1.**
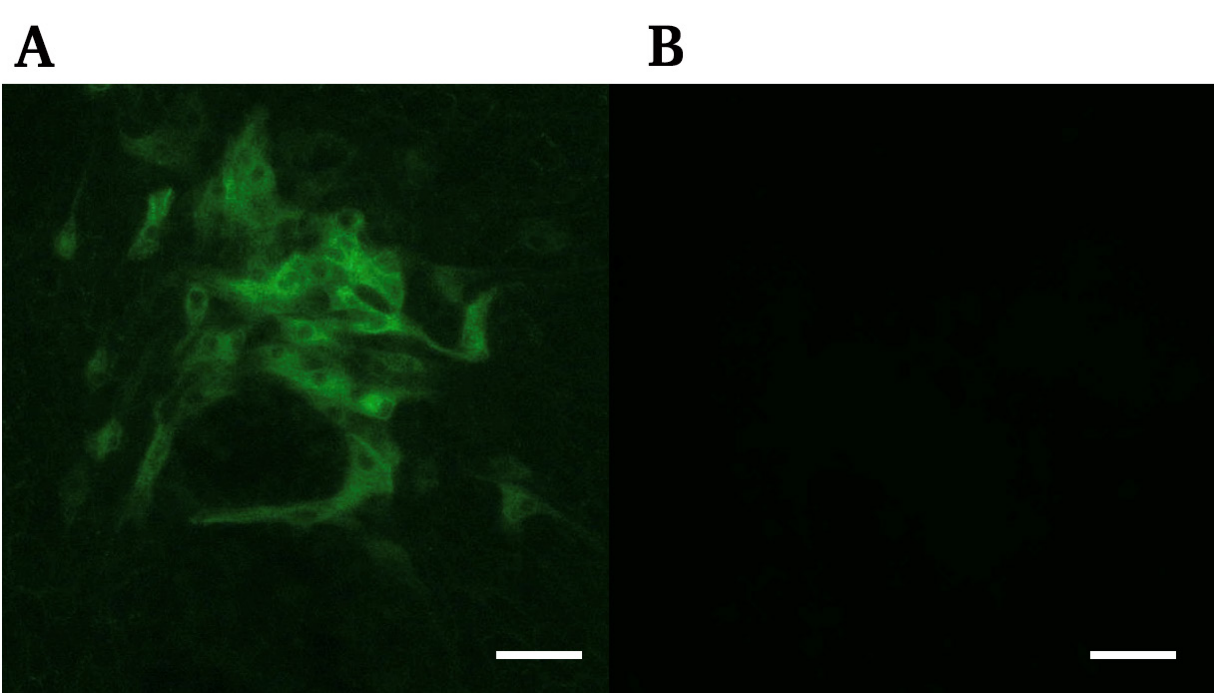
Image of immunofluorescence staining showing bovine viral diarrhea virus (BVDV)-positive cells. Antiviral NS3 immunofluorescence image of infected MDBK cells (A) and non-infectious condition (B). Scale bar, 100 μm.

1. Inoculate newly seeded MDBK cells at 80% confluence in a collagen-coated 24-well microplate with 100 μL of culture supernatant.

2. After 5 days of infection, wash with D-PBS and fix cells with acetone for 10 min at 4 °C. After washing with D-PBS three times, the plate can be stored at 4 °C.

3. Incubate with the indicated anti-pestiviral NS3 antibody (1:1,000 dilution) in D-PBS for 1 h at 37 °C in a humidified atmosphere.

4. After washing with D-PBS three times, incubate cells in the dark with goat anti-mouse IgG Alexa Fluor 488-conjugated (1:1,000 dilution) secondary antibody in D-PBS for 1 h at room temperature.

5. After washing with D-PBS four times, observe under the fluorescence microscope using the BX-Z filter GFP (Figure 1).

## Data analysis

**Figure 2.**
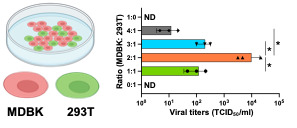
Optimization of the co-culture of two cell lines for the production of recombinant bovine viral diarrhea virus (BVDV). The two cell lines employed in this study were bovine MDBK, which is highly permissive for BVDV infection, and 293T, which shows high transfection efficiency and protein expression. The infectious titers of the prepared working virus were measured by 50% tissue culture infectious dose (TCID_50_) for BVDV. The value of TCID50/mL was calculated using the Reed–Muench method [20]. The ratios of cell numbers and infectious titers are shown as a bar graph. Asterisks indicate significant differences (*p < 0.05) between a pair.

Significant differences in viral titers between different ratios of co-cultures (Figure 2) were analyzed by student’s t-test using GraphPad Prism software.

## Validation of protocol

This protocol, or parts of it, have been applied and validated in the following research article:

• Tamura et al. [13]. A rapid and versatile reverse genetics approach for generating recombinant positive-strand RNA viruses that use IRES-mediated translation (see Figure 2B, H, I).

## General notes and troubleshooting

**General notes**

1. Viral RNA purified from infected cells or culture supernatants can also be used.

2. Because MDBK cells, which are difficult to transfect, are the primary choice for BVDV research, we employed a co-culture strategy with 293T cells to deliver sufficient viral cDNA into MDBK cells. The 293T monocultures were unable to produce BVDV, but recombinant BVDV was produced in transfected co-cultures with a ratio of 2:1 (MDBK:293T), which was the ratio demonstrating the greatest efficacy (Figure 2).

3. The protocol can also generate HCV, hepatitis A virus (HAV), and encephalomyocarditis virus (EMCV) using primer sets and cells appropriate for each virus.

**Troubleshooting**

Problem 1: Transfection is unsuccessful.

Possible cause: Neighboring fragments are not joined.

Solution: Change the overlapping region or length between neighboring fragments, use PCR assembly to connect neighboring fragments, and assess the size of joined fragments by gel electrophoresis.

Problem 2: PCR for amplifying a total of six viral gene fragments is unsuccessful.

Possible cause: The cDNA amount of the viral genome is insufficient for PCR.

Solution: Purify viral RNA from high-viral serum, infected cells, or culture supernatants.

## References

[1] Venter J. C., Glass J. I., Hutchison C. A. and Vashee S. (2022). Synthetic chromosomes, genomes, viruses, and cells. Cell. 185(15): 2708 2724 2724. 10.1016/j.cell.2022.06.046 35868275 PMC9347161

[2] F. Almazan, DeDiego M. L., C. Galan, Escors D., E. Alvarez, Ortego J., Sola I., S. Zuniga, Alonso S., Moreno J. L., .(2006). Construction of a Severe Acute Respiratory Syndrome Coronavirus Infectious cDNA Clone and a Replicon To Study Coronavirus RNA Synthesis. J Virol. 80(21): 10900 10906 10906. 10.1128/jvi.00385-06 16928748 PMC1641757

[3] Yount B., Curtis K. M., Fritz E. A., Hensley L. E., Jahrling P. B., Prentice E., Denison M. R., Geisbert T. W. and Baric R. S. (2003). Reverse genetics with a full-length infectious cDNA of severe acute respiratory syndrome coronavirus. Proc Natl Acad Sci USA. 100(22): 12995 13000 13000. 10.1073/pnas.1735582100 14569023 PMC240733

[4] Scobey T., Yount B. L., Sims A. C., Donaldson E. F., Agnihothram S. S., Menachery V. D., Graham R. L., Swanstrom J., Bove P. F., Kim J. D., .(2013). Reverse genetics with a full-length infectious cDNA of the Middle East respiratory syndrome coronavirus. Proc Natl Acad Sci USA. 110(40): 16157 16162 16162. 10.1073/pnas.1311542110 24043791 PMC3791741

[5] Terada Y., Kuroda Y., Morikawa S., Matsuura Y., Maeda K. and Kamitani W. (2019). Establishment of a Virulent Full-Length cDNA Clone for Type I Feline Coronavirus Strain C3663. J Virol. 93(21): e01208–19. https://doi.org/10.1128/jvi.01208-19 PMC6803248 31375588

[6] Edmonds J., van Grinsven E., Prow N., Bosco-Lauth A., Brault A. C., Bowen R. A., Hall R. A. and Khromykh A. A. (2013). A Novel Bacterium-Free Method for Generation of Flavivirus Infectious DNA by Circular Polymerase Extension Reaction Allows Accurate Recapitulation of Viral Heterogeneity. J Virol. 87(4): 2367 2372 2372. 10.1128/jvi.03162-12 23236063 PMC3571472

[7] Tamura T., Fukuhara T., Uchida T., Ono C., Mori H., Sato A., Fauzyah Y., Okamoto T., Kurosu T., Setoh Y. X., .(2018). Characterization of Recombinant Flaviviridae Viruses Possessing a Small Reporter Tag. J Virol. 92(2): e01582–17. https://doi.org/10.1128/jvi.01582-17 PMC5752933 29093094

[8] Motozono C., Toyoda M., Zahradnik J., Saito A., Nasser H., Tan T. S., Ngare I., Kimura I., Uriu K., Kosugi Y., .(2021). SARS-CoV-2 spike L452R variant evades cellular immunity and increases infectivity. Cell Host Microbe. 29(7): 1124 1136 1136 .e11. 10.1016/j.chom.2021.06.006 34171266 PMC8205251

[9] Saito A., Irie T., Suzuki R., Maemura T., Nasser H., Uriu K., Kosugi Y., Shirakawa K., Sadamasu K., Kimura I., .(2022). Enhanced fusogenicity and pathogenicity of SARS-CoV-2 Delta P681R mutation. Nature. 602(7896): 300–306. 10.1038/s41586-021-04266-9

[10] Tamura T., Ito J., Uriu K., Zahradnik J., Kida I., Anraku Y., Nasser H., Shofa M., Oda Y., Lytras S., .(2023). Virological characteristics of the SARS-CoV-2 XBB variant derived from recombination of two Omicron subvariants. Nat Commun. 14(1): 2800 10.1038/s41467-023-38435-3 37193706 PMC10187524

[11] Tamura T., Irie T., Deguchi S., Yajima H., Tsuda M., Nasser H., Mizuma K., Plianchaisuk A., Suzuki S., Uriu K., .(2024). Virological characteristics of the SARS-CoV-2 Omicron XBB.1.5 variant. Nat Commun. 15(1): 1176 10.1038/s41467-024-45274-3 38332154 PMC10853506

[12] Tamura T., Mizuma K., Nasser H., Deguchi S., Padilla-Blanco M., Oda Y., Uriu K., Tolentino J. E., Tsujino S., Suzuki R., .(2024). Virological characteristics of the SARS-CoV-2 BA.2.86 variant. Cell Host Microbe. 32(2): 170 180 180 .e12. 10.1016/j.chom.2024.01.001 38280382

[13] Tamura T., Yamamoto H., Ogino S., Morioka Y., Tsujino S., Suzuki R., Hiono T., Suzuki S., Isoda N., Sakoda Y., .(2024). A rapid and versatile reverse genetics approach for generating recombinant positive-strand RNA viruses that use IRES-mediated translation. J Virol. 98(3): e01638–23. https://doi.org/10.1128/jvi.01638-23 PMC10949505 38353536

[14] Ruggli N. and Rice C. M. (1999). Functional cDNA Clones of The Flaviviridae: Strategies and Applications. Adv Virus Res. 53: 183 207 207. 10.1016/s0065-3527(08)60348-6 10582099

[15] Nagai M., Sakoda Y., Mori M., Hayashi M., Kida H. and Akashi H. (2003). Insertion of cellular sequence and RNA recombination in the structural protein coding region of cytopathogenic bovine viral diarrhoea virus. J Gen Virol. 84(2): 447 452 452. 10.1099/vir.0 .18773-0 12560578

[16] Osorio J. S. and Bionaz M. (2017). Plasmid transfection in bovine cells: Optimization using a realtime monitoring of green fluorescent protein and effect on gene reporter assay. Gene. 626: 200 208 208. 10.1016/j.gene.2017.05.025 28501631

[17] Hirose S., Notsu K., Ito S., Sakoda Y. and Isoda N. (2021). Transmission Dynamics of Bovine Viral Diarrhea Virus in Hokkaido, Japan by Phylogenetic and Epidemiological Network Approaches. Pathogens. 10(8): 922 10.3390/pathogens10080922 34451386 PMC8400418

[18] Kameyama K., Sakoda Y., Tamai K., Igarashi H., Tajima M., Mochizuki T., Namba Y. and Kida H. (2006). Development of an immunochromatographic test kit for rapid detection of bovine viral diarrhea virus antigen. J Virol Methods. 138: 140 146 146. 10.1016/j.jviromet.2006.08.005 17046073

[19] Masaki T., Suzuki R., Saeed M., Mori K. I., Matsuda M., Aizaki H., Ishii K., Maki N., Miyamura T., Matsuura Y., .(2010). Production of Infectious Hepatitis C Virus by Using RNA Polymerase I-Mediated Transcription. J Virol. 84(11): 5824 5835 5835. 10.1128/jvi.02397-09 20237083 PMC2876618

[20] Reed L. and Muench H. (1938). A Simple Method Of Estimating Fifty Per Cent Endpoints12. Am J Epidemiol. 27(3): 93 497 497. 10.1093/oxfordjournals.aje.a118408

